# Radiographic progression based on baseline characteristics from TNF inhibitor biosimilar studies in patients with rheumatoid arthritis

**DOI:** 10.1186/s13075-020-02267-z

**Published:** 2020-08-14

**Authors:** Josef S. Smolen, Young Mo Kang, Wan-Hee Yoo, Paul Emery, Michael E. Weinblatt, Edward C. Keystone, Mark C. Genovese, Gihyun Myung, Inyoung Baek, Jeehoon Ghil

**Affiliations:** 1 Division of Rheumatology, Department of Medicine, Medical University of Vienna, Waehinger Guertel 18-20, A-1090 Vienna, Austria; 2 Division of Rheumatology, Department of Internal Medicine, Kyungpook National University School of Medicine, Daegu, Republic of Korea; 3 Division of Rheumatology, Department of Internal Medicine, Chonbuk National University Hospital, Jeonju, Republic of Korea; 4 University of Leeds, Leeds Institute of Rheumatic and Musculoskeletal Medicine, Leeds, UK; 5 Division of Rheumatology, Immunology, and Allergy, Brigham and Women’s Hospital, Harvard Medical School, Boston, MA USA; 6 Division of Rheumatology, Mount Sinai Hospital, University of Toronto, Toronto, Ontario Canada; 7 Division of Immunology and Rheumatology, Stanford University Medical Center, Stanford, CA USA; 8 Samsung Bioepis Co., Ltd., Incheon, Republic of Korea

**Keywords:** Biosimilar, TNF inhibitors, Rheumatoid arthritis, Radiography

## Abstract

**Objective:**

Phase III clinical trials of the tumour necrosis factor inhibitors SB4, SB2, and SB5 (biosimilars to etanercept, infliximab, and adalimumab, respectively) have demonstrated efficacy in moderate-to-severe rheumatoid arthritis (RA). Data from these trials were used to identify baseline characteristics associated with radiographic progression and to build a matrix risk model for its prediction.

**Methods:**

Patients with radiographic progression and baseline demographic and disease characteristic data were pooled across the 3 phase III studies of each biosimilar and its reference product. Baseline demographics and disease characteristics were evaluated for their relationship with radiographic progression (1-year mean change in mTSS > 0); 3 factors were selected based on strongest Pearson’s correlation coefficient with the change in modified Total Sharp Score. Univariate logistic regression was performed to assess the association between each baseline factor and the rate of radiographic progression, with subsequent matrix model development performed using multivariate logistic regression.

**Results:**

A total of 1371 patients were included in the analysis, with a radiographic progression rate of 27.4%. The 3 baseline predictors of radiographic progression, based on Pearson’s correlation coefficient, were 28 swollen joint count (SJC28), C-reactive protein (CRP), and physician global assessment (PhGA). A matrix model showed that the predicted risk of radiographic progression was higher with the increased level of SJC28, CRP, and PhGA (*P* < 0.001).

**Conclusions:**

In this pooled analysis of phase III clinical trial data of biosimilars for RA, identifiable baseline factors (SJC28, CRP, and PhGA) associated with radiographic progression were similar to those described in prior studies. Even though radiographic progression was minimal, a small number of patients who have increased SJC28, CRP, and PhGA at baseline should be closely monitored and follow treat-to-target approach.

**Clinical trial registration numbers:**

EudraCT 2012-005026-30. Registered 30 April 2013, https://www.clinicaltrialsregister.eu/ctr-search/trial/2012-005026-30/results

EudraCT 2012-005733-37. Registered 10 July 2013, https://www.clinicaltrialsregister.eu/ctr-search/trial/2012-005733-37/results

EudraCT 2013-005013-13. Registered 01 April 2014, https://www.clinicaltrialsregister.eu/ctr-search/trial/2013-005013-13/results

## Introduction

Rheumatoid arthritis (RA) is a chronic, systemic disorder that causes clinical symptoms as well as structural joint damage leading to functional disability, poor quality of life, decreased work productivity, and substantial societal cost in terms of both direct and indirect costs [1–5]. Achievement of remission or low disease activity remains the overarching goal of therapy, regardless of whether patients have early or established RA, with therapeutic decisions guided by the extent of disease activity and prognostic factors [6–8]. Reducing joint damage progression, as visualised by radiographic changes, is important given its correlation with irreversible functional impairment [9, 10]. The biologic disease-modifying anti-rheumatic drugs (bDMARDs) etanercept, infliximab, and adalimumab are tumour necrosis factor (TNF) inhibitors for which RA is among their approved indications [11–13]. Randomised clinical trials of these agents in patients with RA have collectively and consistently demonstrated benefits with respect to reducing disease activity, inhibiting radiographic progression, and inducing clinical remission, with significant advantages for combination therapy versus monotherapy with methotrexate (MTX) or a TNF inhibitor alone [14–24]. Study extension data are also available and have demonstrated the long-term efficacy and safety of TNF inhibitors [25, 26].

Research efforts have identified baseline factors that are predictive of radiographic progression and provide clinical value for identifying patients who require intensive treatment and monitoring from the time of diagnosis. Prior studies of nonbiologic and bDMARDs have revealed certain baseline factors associated with radiographic progression, including baseline swollen joint count (SJC), C-reactive protein (CRP) level, erythrocyte sedimentation rate (ESR), rheumatoid factor (RF) and anti-citrullinated peptide antibody (ACPA) status, and the presence of erosions [27–29]. Disease activity, as evaluated by composite indices, has also been found to have a strong association with progression of joint damage [12, 14, 15, 30], though the use of bDMARDs may result in less radiographic progression than MTX alone across different disease activity spectrums [15, 31–36].

Risk factors for rapid radiographic progression have been identified in terms of a matrix risk model more than a decade ago [28] and since then have been confirmed [27, 29, 37]. However, these matrix models looked at the risk of patients who were either on placebo with conventional synthetic DMARDs (csDMARDs) like MTX as background therapy or on newly administered MTX and compared this with progression in patients treated with bDMARDs plus MTX. Because radiographic progression is quite intensive with csDMARDs or placebo and progression rates were much higher in earlier years [38], researchers primarily looked at rapid radiographic progression which was defined as an annual progression rate of ≥ 5 modified Sharp/van der Heijde score points per year [28, 29]. However, using this definition can be difficult for patients on anti-TNFs because only a small number of them have such large radiographic progression rates while on anti-TNFs, aside from less progression seen in recent years.

Several previous studies showed a blunted relationship between the progression of joint damage and clinical disease activity during the course of treatment with bDMARDs [15, 31–36], and it has not been clarified yet whether bDMARDs reduce or halt progression of damage irrespective of disease activity or there is still a strict relationship to clinical activity. Moreover, having no progression of joint damage is an important aspect, since it provides evidence for a full abrogation, and not just mitigation, of the structural aggressiveness of RA. In the present study, we used a large database collectively derived across the phase III clinical trials of biosimilars SB4, SB2, and SB5 and the reference products etanercept, infliximab, and adalimumab, thus encompassing 3 different TNF-inhibitors to look at whether there are baseline measures that reflect disease activity associated with radiographic progression.

## Methods

### Data sources

This is a pooled analysis of 3 phase III studies that compared the efficacy and safety of each biosimilar TNF inhibitor (SB4, SB2, and SB5) with its associated reference product (etanercept, infliximab, and adalimumab, respectively) [39–41]. All 3 clinical studies were multicentre, randomised, double-blind, and parallel-group in design and enrolled patients with moderately or severely active RA despite MTX treatment, conducted to evaluate the efficacy, pharmacokinetics, safety, and immunogenicity of the biosimilar in comparison with its reference product in these patients.

The methodologies of each of the 3 studies have been published in detail elsewhere [39–41]. The eligibility criteria for these 3 studies were similar, resulting in similar patient demographics across studies. In brief, patients were aged 18 to 75 years and had been diagnosed with RA (per American College of Rheumatology 1987 revised classification criteria, in accordance with the respective comparator trials performed with the original agents); all patients had a disease duration of ≥ 6 months, during which they had received MTX for ≥ 6 months and at a stable dosage for ≥ 4 weeks before screening or randomisation. Additional requirements included the presence of active disease as evidenced by ≥ 6 swollen joints and ≥ 6 tender joints and either ESR ≥ 28 mm/h or serum CRP level ≥ 1.0 mg/dL. No prior treatment with biologic agents was allowed.

Since the effects of all 3 TNF inhibitors on radiographic progression were similar and were also shown to be similar between originator and biosimilar TNF blockers [42–44], the data of all trial arms were pooled and included all patients who had baseline demographic and disease characteristic data available, as well as radiographic results at baseline and study end (week 52 for SB4/etanercept and SB5/adalimumab studies, week 54 for the SB2/infliximab study).

### Data extraction

Structural joint damage was assessed from x-rays of both hands and feet. Radiographs were scored at week 0 and the final study week (week 52 for SB4/etanercept and SB5/adalimumab studies, week 54 for the SB2/infliximab study) using the modified Total Sharp Score (mTSS), the sum of the joint erosion score and the joint space narrowing score [45, 46]. X-rays from week 0 and the final study week were scored centrally by 2 independent qualified individuals under blinded conditions. The mean score for the change in mTSS from the 2 assessments was used for the analysis. Radiographic progression was defined as a 1-year mean change in mTSS > 0.

### Data analysis

Patients with radiographic data, baseline demographics, and disease characteristics available from each study were pooled and analysed. The 3 individual baseline factors most associated with radiographic progression were identified based on those having the strongest Pearson’s correlation coefficient with the change in mTSS. Various demographic (e.g., age and sex) and disease characteristics (e.g., baseline SJC28, CRP, ESR, and RF positivity) were analysed (see Table 1 for complete list); composite scores (e.g., Simplified Disease Activity Index [SDAI], and Clinical Disease Activity Index [CDAI]) were also analysed, but not selected for baseline factors included in the matrix. We did not primarily employ Disease Activity Score in 28 joints [DAS28] by ESR, since this score should not anymore be used to define remission according to ACR-EULAR remission definitions and newer insights [47, 48]. Univariate logistic regression was performed to assess the association between each baseline factor and the proportion of patients with radiographic progression. Multivariate logistic regression was used to develop the matrix model of the 3 identified individual baseline factors to show the proportion of patients with radiographic progression in trichotomised cutoffs of each baseline factor.

**Table 1:** Baseline demographic and disease characteristics

Characteristic^**a**^	Total number of patients ***N*** = 1371	Correlation coefficient	***P*** value of Pearson’s correlation coefficient with the change in mTSS
Baseline mTSS	37.9 (56.8)	0.025	0.36
Age, years	51.4 (12.0)	− 0.023	0.39
Female, *n* (%)	1120 (81.7)	0.032	0.24
RA disease duration, years	5.9 (4.9)	0.0019	0.94
MTX dose, mg/week	15.2 (4.4)	− 0.0021	0.94
SJC28	11.0 (5.1)	0.077	0.0041
TJC28	15.1 (6.3)	0.041	0.13
HAQ-DI	1.5 (0.6)	0.049	0.071
DAS28 (ESR)	6.5 (0.8)	N/A	0.022
SDAI	39.9 (11.8)	N/A	0.004
CDAI	38.5 (11.4)	N/A	0.009
PhGA VAS, mm	61.9 (15.5)	0.054	0.048
Patient global assessment VAS, mm	61.9 (18.6)	0.041	0.13
Patient pain assessment VAS, mm	62.0 (19.5)	0.043	0.11
CRP, mg/L	13.3 (19.1)	0.057	0.033
ESR, mm/h	44.4 (20.4)	0.042	0.12
RF positive,^b^ *n* (%)	1033 (75.4)	0.024	0.73

*CDAI* Clinical Disease Activity Index, *CRP* C-reactive protein, *DAS28* disease activity score by 28 joint count, *ESR* erythrocyte sedimentation rate, *HAQ-DI* health assessment questionnaire disability index, *mTSS* modified Total Sharp Score, *MTX* methotrexate, *N/A* not applicable, *PhGA* physician global assessment, *RA* rheumatoid arthritis, *RF* rheumatoid factor, *SDAI* Simplified Disease Activity Index, *SJC28* 28 swollen joint count, *TJC28* 28 tender joint count, *VAS* visual analogue scale

^a^Data are presented as mean (SD), unless indicated otherwise

^b^RF positive indicates > 14 kIU/L for SB4, SB2, and SB5

In exploratory analyses, further multivariate logistic analyses were performed based on the identified 3 baseline factors using the same dataset to predict the proportion of patients in remission or low disease activity (LDA) by CDAI, SDAI, and DAS28 at week 24/30. Separate matrices were built on joint space narrowing and joint erosion score, subcomponents of mTSS, and for the proportion of patients with joint erosion score > 0 and joint space narrowing > 0 in trichotomised cutoffs of each baseline factor.

## Results

Overall, the analysis included 1371 patients, 376 (27.4%) of whom experienced radiographic progression (> 0) with a mean change in mTSS of 0.41 (SD, 3.21). Of note, while on TNF inhibitor therapy, 121 (8.8%) patients had progression of mTSS ≥ 3. Baseline characteristics for all patients are displayed in Table 1. For all treatments combined, the mean age of participants was 51.4 years and most patients were female (81.7%). The mean duration of RA was 5.9 years, and mean mTSS was 37.9 at baseline. Based on Pearson’s correlation coefficient, the SJC28, CRP, and physician global assessment (PhGA) were determined to be significant baseline predictors of radiographic progression and used for further analysis (Table 1). DAS28, SDAI, and CDAI were also significant with *P* values < 0.05 (Table 1).

Although RF status (positive [> 14 kIU/L] vs negative) significantly correlated with the presence of radiographic changes (yes/no) (*P* = 0.002), it did not significantly correlate with the numerical change in mTSS, which was the measurement used to extract individual baseline factors that were most associated based on Pearson’s correlation coefficient (*P* = 0.7261).

**Fig. 1:**
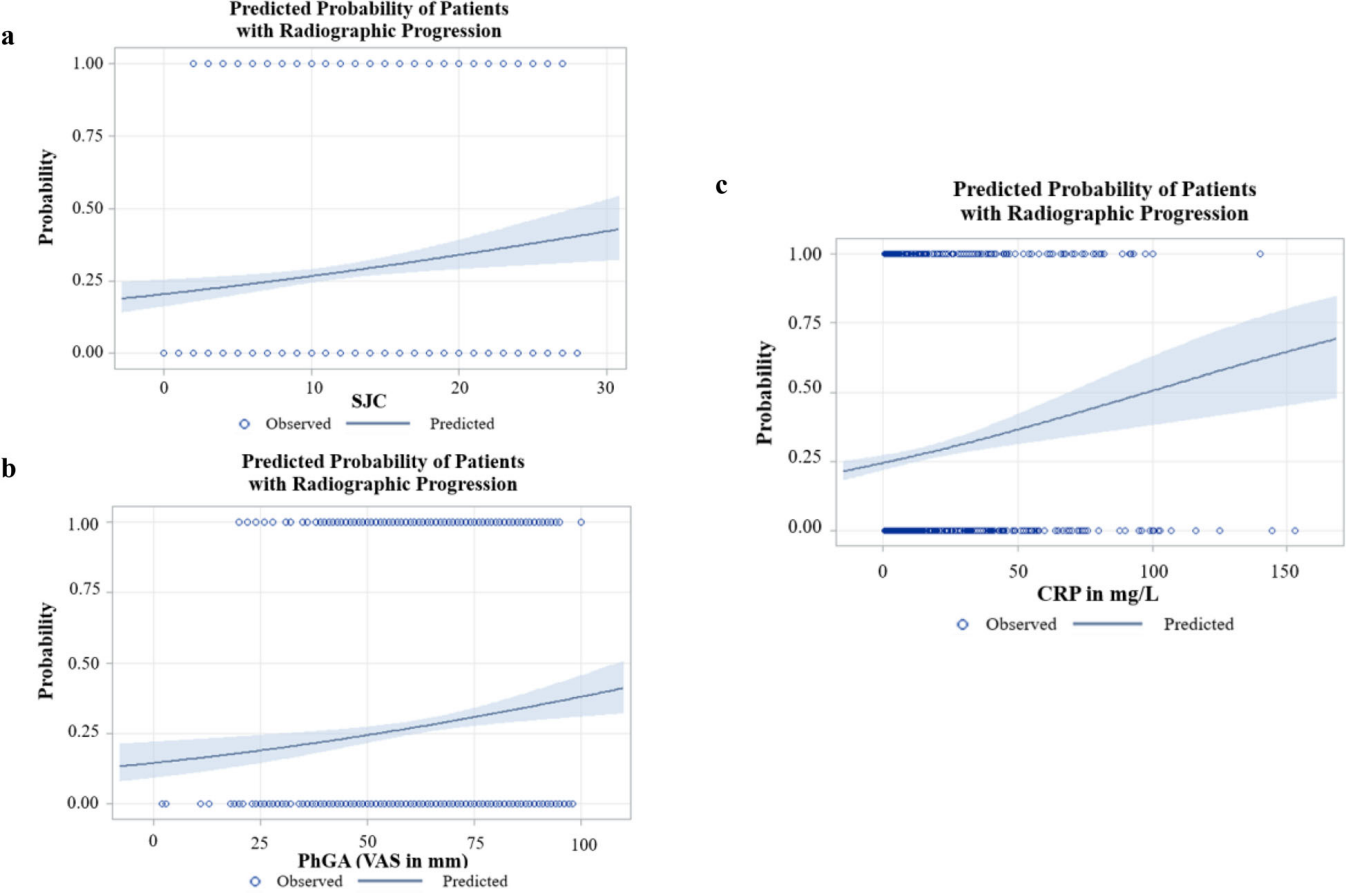
**a**–**c** Predicted probability (with 95% confidence limits) of patients with radiographic progression (yes/no) based on **a** SJC28, **b** PhGA, and **c** CRP. CRP, C-reactive protein; PhGA, physician global assessment; SJC28, 28 swollen joint count

The predicted risk of radiographic progression as a function of SJC28, CRP, and PhGA in the all treatments combined group is shown in Fig. 1a–c. Overall, as SJC28, CRP, and PhGA increased, the predicted risk of radiographic progression likewise increased. A similar pattern was shown for CDAI and SDAI (Supplementary Fig. 1A, B).

**Fig. 2:**
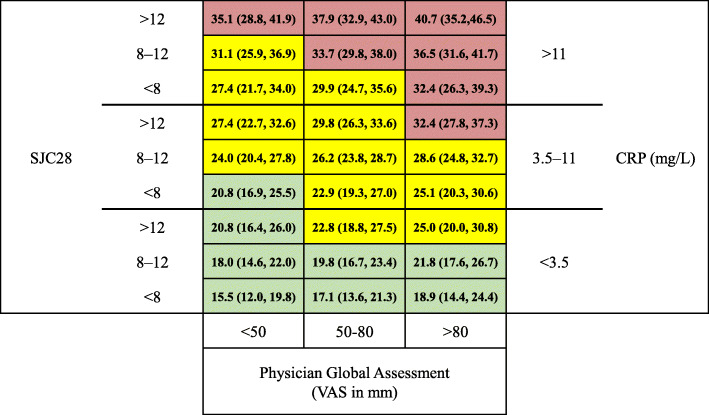
Matrix model of the proportion of patients with radiographic progression based on SJC28, CRP, and PhGA. Data are presented as % (95% confidence interval). Predicted probability of patients with radiographic progression represented by shading: green, < 22.0%; yellow, 22.0–32.0%; red, > 32.0%. CRP, C-reactive protein; SJC28, 28 swollen joint count; PhGA, physician global assessment; VAS, visual analogue scale

Development of the matrix risk model on radiographic progression (Fig. 2) showed that the 3 risk factors were associated with radiographic progression (*P* < 0.001). The predicted proportion of patients with radiographic progression tended to increase as the matrix moved from the lower to the higher range of each risk factor. As an example, the proportion of radiographic progressors (patients with the 1-year change in mTSS > 0) in the group with the highest cutoff value of SJC28, CRP, and PhGA was 40.7% (95% CI, 35.2%, 46.5%), whereas the proportion of radiographic progressors in the lowest cutoff value of SJC28, CRP, and PhGA was 15.5% (95% CI, 12.0%, 19.8%). In the matrix, CRP had a significant association with radiographic progression (*P* < 0.0001). *P* values for SJC28 and the PhGA were 0.139 and 0.140, respectively.

**Fig. 3:**
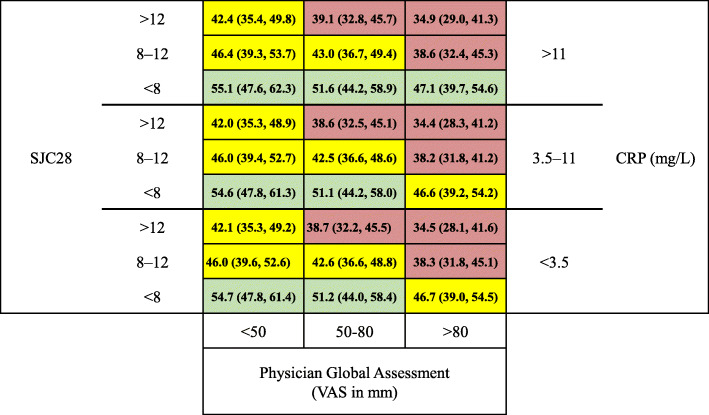
Matrix model of the proportion of patients in remission or low disease activity by CDAI at week 24/30 based on baseline SJC28, CRP, and PhGA. Data are presented as % (95% confidence interval). Predicted probability of patients in remission or low disease activity represented by shading: green, > 47.0%; yellow, 40.0–47.0%; red, < 40.0%. CDAI, Clinical Disease Activity Index; CRP, C-reactive protein; PhGA, physician global assessment; SJC28, 28 swollen joint count; VAS, visual analogue scale

In the exploratory analysis, the matrix for the subcomponents of mTSS score was developed, and it showed that the predicted probability of patients with joint erosion score > 0 or joint space narrowing > 0 increased as each baseline factor worsened (Supplementary Fig. 2A, B). Additional analysis was done by disease activity, and the proportion of patients in remission or LDA by CDAI at week 24/30 tended to decrease with higher levels of baseline SJC28, CRP, and PhGA scores (Fig. 3). The proportion of patients in remission or LDA was 34.9% (95% CI, 29.0%, 41.3%) in the highest baseline cutoff value (SJC28 > 12, CRP > 11 mg/L, and PhGA > 80 mm) and 54.7% (95% CI, 47.8%, 61.4%) in the lowest baseline cutoff value (SJC28 < 8, CRP < 3.5 mg/L, and PhGA < 50 mm). Additionally, the proportion of patients in remission or LDA by the SDAI and DAS28 showed a similar pattern (Supplementary Fig. 3A, B). The total numbers of patients in each tertile of the 3 baseline factors in the matrix model are shown in Supplementary Fig. 4.

## Discussion

In this pooled study of 1371 patients from the SB4/etanercept, SB2/infliximab, and SB5/adalimumab phase III studies of biosimilars and originators for the treatment of RA, radiographic progression, although present, was minimal overall except a small proportion of patients with progression. Notably, studies involving conventional synthetic DMARDs have shown more significant radiographic progression [27–29]. Despite using higher mTSS cutoff values in those studies, the proportion of patients with radiographic progression was higher, which reflects the effectiveness of TNF inhibitors in slowing radiographic progression. Indeed, in previous studies, the effects on rapid radiographic progression were assessed [27–29], whereas in this study, we focused on prediction of any damage progression (mean change in mTSS > 0) on TNF inhibitors.

Overall, the findings of the current analyses were consistent with those from previous studies based on conventional DMARDs or reference biologics in identifying CRP levels and SJC28 as significant baseline factors associated with radiographic progression in patients receiving MTX or other csDMARDs [27–29, 37] while RF levels were not. The use of bDMARDs is known to blunt the correlation between the baseline disease activities and radiographic progression [15, 31–33, 35]. Nevertheless, we observed that baseline CDAI and SDAI still showed a relationship with the change in mTSS based on Pearson’s correlation coefficient (*P* value 0.004 for SDAI and 0.009 for CDAI) and predicted probability of patients with radiographic progression (yes/no) based on SDAI and CDAI (Supplementary Fig. 1A, B). This is in line with the data obtained from assessing the individual components of these composite measures, since it is postulated from our study that the correlation is due to CRP, SJC28, and to some extent also PhGA which is usually a reflection of SJC and CRP [49, 50]. However, the much stronger significance of the association between composite measures and radiographic progression compared with the individual variables once again confirms the advantage of assessing composite scores. Fautrel et al. sought to develop a predictive algorithm and matrix in patients with early RA initiating synthetic DMARD therapy using SJC, CRP level, RF or ACPA status, and the presence of typical RA erosions as predictors of radiographic progression that were applied in the final multivariate model [27]. Data from clinical trials in which some patients received infliximab were the first used in the development of risk models for rapid radiographic progression [28, 29]. More specifically, matrix modelling based on the ASPIRE study of MTX alone or with infliximab in MTX-naive early RA was developed using SJC28, RF, and either CRP or ESR (with the goal of making the model interchangeable from a clinical practice standpoint), with treatment arm as a dichotomous variable [28]. It showed a dramatic structural advantage of treating patients with a TNFi+MTX vs MTX alone regarding the risk of rapid radiographic progression. When that model was expanded to another study of infliximab (the ATTRACT study of continued MTX with infliximab or placebo in patients with active RA despite stable-dose MTX), the use of the combination was again associated with a low risk of rapid radiographic progression when patients were in the low or intermediate ranges of the baseline risk factors, with high risk limited to those patients at the highest baseline risk ranges. Unlike patients on combination therapy, for patients on MTX monotherapy, risk of rapid radiographic progression was high irrespective of baseline risk factors.

Subsequently, a matrix risk model developed in a post hoc analysis of the BeSt study (evaluating MTX-based therapy, including MTX plus infliximab, for recent-onset RA) confirmed these data by identifying baseline CRP level, RF/ACPA status, and in addition erosion score as independent factors for predicting rapid radiographic progression, defined as an increase in Sharp-van der Heijde score ≥ 5 after 1 year [29].

Thus, matrix modelling demonstrated risk reduction with initial combination therapy that included infliximab or prednisone relative to MTX monotherapy, establishing treatment choice as a main determinant of rapid radiographic progression. However, in contrast to these prior risk models that determined risk by treatment arm, the current analysis did not compare radiographic progression by product or between biosimilars and reference products.

The goal of our study was to determine if one could discern patients at risk for progression of joint damage while on TNF inhibitors, since joint damage is generally a sign of aggressive disease and high inflammatory propensity, especially if cumulative over time.

Therefore, we did not examine rapid radiographic progression, since it is primarily observed upon administration of conventional synthetic DMARDs or placebo. However, since the matrix model shows that TNF inhibitors cannot prevent progression in patients at high risk, such patients may have to receive another treatment than a TNF-blocker, given their significant progression on these drugs. The predicted model also showed that patients with high levels of SJC28, CRP, and PhGA at baseline also showed slightly increased risk of higher disease activity by CDAI at week 24/30. Based on this, one can infer that baseline characteristics have a relationship with radiographic progression and disease activity. This further supports the importance of the treat-to-target approach; if the treatment target of LDA is not met between weeks 12 to 24, advancing to the next line of therapy is recommended [7]. We also demonstrated an association between PhGA and radiographic progression in the setting of RA. In our study, RF levels were not found to be associated with the change in mTSS at 1-year and, therefore, it did not become part of the matrix model. This is likely explained by the fact that anti-TNF blunts the association between RF status and radiographic progression [51], unlike in the studies on conventional synthetic DMARDs. Of note, the presence of RF determines disease activity and, therefore, RF appears to primarily act via increasing inflammation [52–54]. The ACPA status was not available and not included in the analysis.

This analysis has several limitations. Most notably, the lack of a placebo or MTX monotherapy group in the phase III studies precludes prediction of radiographic progression in reference to no biologic treatment. Another limitation is that ACPA status was not obtained in the study. Additionally, although the phase III studies had similar designs, patient demographics, and disease characteristics, they were not primarily designed to be combined.

To identify baseline factors for patients on anti-TNFs, a more stringent definition of radiographic progression compared to previous matrix models [28, 29] was employed to reveal a halt, and not just a reduction of damage progression.

## Conclusions

Our analysis of phase III clinical trials of biosimilars and reference products for RA identified baseline factors that were consistent with those identified for rapid radiographic progression in previous studies of csDMARD therapy in RA. This signifies that even though radiographic progression is minimal while on anti-TNF, a small proportion of patients still has significant progression, and clinicians should closely monitor patients who have high SJC28, CRP, and PhGA before treatment start whether they are started on csDMARDs or anti-TNFs.

## Supplementary information

**Additional file 1 : Table S1.** Disease activity cut-off values. **Fig. S1A.** Predicted probability (with 95% confidence limits) of patients with radiographic progression (yes/no) based on CDAI. **B.** Predicted probability (with 95% confidence limits) of patients with radiographic progression (yes/no) based on SDAI. **Fig. S2A.** Matrix model of the proportion of patients with joint erosion score > 0 based on SJC28, CRP, and PhGA. Data are presented as % (95% confidence interval). Predicted probability of patients represented by shading: green, < 20.0%; yellow, 20.0–25.0%; red, > 25.0%. **B.** Matrix model of the proportion of patients with joint space narrowing > 0 based on SJC28, CRP, and PhGA. Data are presented as % (95% confidence interval). Predicted probability of patients represented by shading: green, < 15.0%; yellow, 15.0–21.0%; red, > 21.0%. **Fig. S3A.** Matrix model of the proportion of patients in remission or low disease activity by SDAI at week 24/30 based on SJC28, CRP, and PhGA. Data are presented as % (95% confidence interval). Predicted probability of patients in remission with low disease activity represented by shading: green, > 47.0%; yellow, 40.0–47.0%; red, < 40.0%. **B.** Matrix model of the proportion of patients in remission or with low disease activity by DAS28 at week 24/30 based on SJC28, CRP, and PhGA. Data are presented as % (95% confidence interval). Predicted probability of patients in remission with low disease activity represented by shading: green > 32.0%; yellow, 27.0–32.0%; red, < 27.0%. **Fig. S4.** Number of patients corresponding to each tertile of the 3 baseline factors.

## Data Availability

Upon request, and subject to certain criteria, conditions, and exceptions, Samsung Bioepis will provide access to individual de-identified participant data. The de-identified participant data will be made available to researchers whose proposals meet the research criteria and other conditions, and for which an exception does not apply. The proposals should be directed to the corresponding author. To gain access, data requestors must enter into a data access agreement with Samsung Bioepis.
