# Supplementary material for: Radiographic progression based on baseline characteristics from TNF inhibitor biosimilar studies in patients with rheumatoid arthritis
Source: Arthritis Res Ther. 2020 Aug 14;22:188. doi: 10.1186/s13075-020-02267-z (PMC7427775; doi:10.1186/s13075-020-02267-z)
Supplement: Supplementary file 1 — Additional file 1 : Table S1. Disease activity cut-off values. Fig. S1A. Predicted probability (with 95% confidence limits) of patients with radiographic progression (yes/no) based on CDAI. B. Predicted probability (with 95% confidence limits) of patients with radiographic progression (yes/no) based on SDAI. Fig. S2A. Matrix model of the proportion of patients with joint erosion score > 0 based on SJC28, CRP, and PhGA. Data are presented as % (95% confidence interval). Predicted probability of patients represented by shading: green, < 20.0%; yellow, 20.0–25.0%; red, > 25.0%. B. Matrix model of the proportion of patients with joint space narrowing > 0 based on SJC28, CRP, and PhGA. Data are presented as % (95% confidence interval). Predicted probability of patients represented by shading: green, < 15.0%; yellow, 15.0–21.0%; red, > 21.0%. Fig. S3A. Matrix model of the proportion of patients in remission or low disease activity by SDAI at week 24/30 based on SJC28, CRP, and PhGA. Data are presented as % (95% confidence interval). Predicted probability of patients in remission with low disease activity represented by shading: green, > 47.0%; yellow, 40.0–47.0%; red, < 40.0%. B. Matrix model of the proportion of patients in remission or with low disease activity by DAS28 at week 24/30 based on SJC28, CRP, and PhGA. Data are presented as % (95% confidence interval). Predicted probability of patients in remission with low disease activity represented by shading: green > 32.0%; yellow, 27.0–32.0%; red, < 27.0%. Fig. S4. Number of patients corresponding to each tertile of the 3 baseline factors. [file 13075_2020_2267_MOESM1_ESM.docx]

**Supplementary Table and Figures**

**Supplementary Table 1.** Disease activity cut-off values

| **Disease activity measure** | **Remission** | **LDA** | **MDA** | **HDA** |
| --- | --- | --- | --- | --- |
| **CDAI** | ≤ 2.8 | > 2.8 to ≤ 10.0 | > 10.0 to ≤ 22.0 | > 22.0 |
| **SDAI** | ≤ 3.3 | > 3.3 to ≤ 11.0 | > 11.0 to ≤ 26.0 | > 26.0 |
| **DAS28 (ESR)** | < 2.6 | ≥ 2.6 to < 3.2 | ≥ 3.2 to ≤ 5.1 | > 5.1 |

CDAI, Clinical Disease Activity Index; DAS28, disease activity score based on 28-joint count; ESR, erythrocyte sedimentation rate; HDA, high disease activity; LDA, low disease activity; MDA, moderate disease activity; SDAI, Simplified Disease Activity Index.

**Supplementary Fig. 1A.** Predicted probability (with 95% confidence limits) of patients with radiographic progression (yes/no) based on CDAI


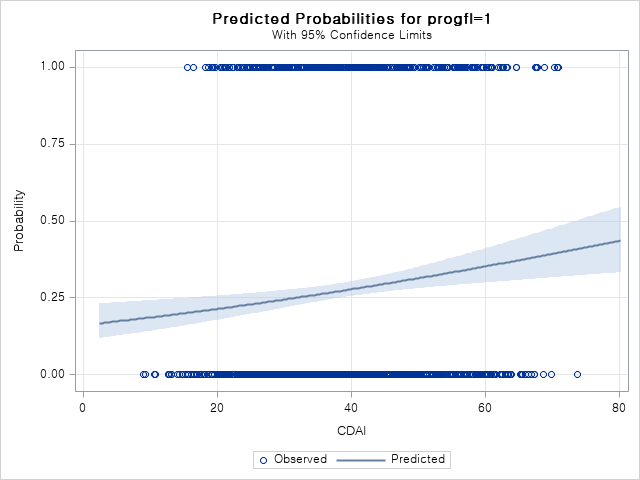


**Predicted Probability of Patients with Radiographic Progression**

**Probability**

**CDAI**

CDAI, Clinical Disease Activity Index

**Supplementary Fig. 1B.** Predicted probability (with 95% confidence limits) of patients with radiographic progression (yes/no) based on SDAI


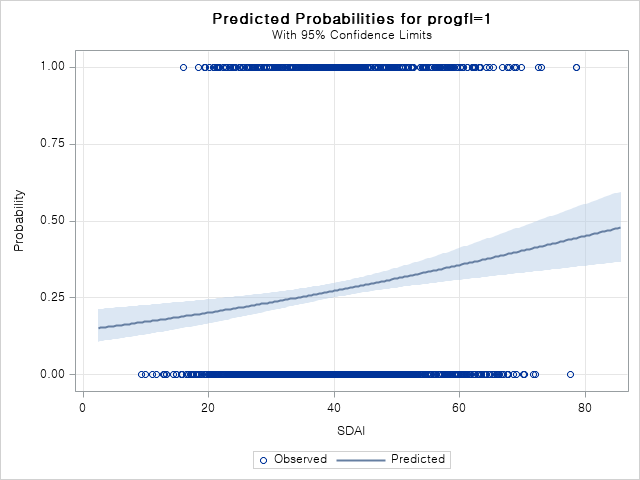


**Probability**

**SDAI**

**Predicted Probability of Patients with Radiographic Progression**

SDAI, Clinical Disease Activity Index

**Supplementary Fig. 2A.** Matrix model of the proportion of patients with joint erosion score >0 based on SJC28, CRP, and PhGA. Data are presented as % (95% confidence interval). Predicted probability of patients represented by shading: green, <20.0%; yellow, 20.0‒25.0%; red, >25.0%.

| SJC28 | >12 | **26.3 (20.6, 32.8)** | **28.3 (23.9, 33.3)** | **30.5 (25.4, 36.1)** | >11 | CRP (mg/L) |
| --- | --- | --- | --- | --- | --- | --- |
|  | 8–12 | **23.8 (19.1, 29.3)** | **25.8 (22.2, 29.8)** | **27.8 (23.3, 32.8)** |  |  |
|  | <8 | **21.6 (16.4, 27.8)** | **23.4 (18.7, 28.8)** | **25.3 (19.7, 31.9)** |  |  |
|  | >12 | **20.3 (16.3, 25.1)** | **22.1 (19.0, 25.6)** | **23.9 (19.9, 28.5)** | 3.5–11 |  |
|  | 8–12 | **18.3 (15.2, 21.9)** | **19.9 (17.8, 22.2)** | **21.6 (18.3, 25.4)** |  |  |
|  | <8 | **16.5 (12.9, 20.7)** | **17.9 (14.7, 21.7)** | **19.5 (15.2, 24.6)** |  |  |
|  | >12 | **15.5 (11.7, 20.2)** | **16.9 (13.4, 21.1)** | **18.4 (14.1, 23.7)** | <3.5 |  |
|  | 8–12 | **13.8 (10.9, 17.5)** | **15.1 (12.4, 18.3)** | **16.5 (12.8, 21.0)** |  |  |
|  | <8 | **12.4 (9.3, 16.3)** | **13.5 (10.4, 17.4)** | **14.8 (10.8, 19.9)** |  |  |
|  | | <50 | 50–80 | >80 |  | |
|  | | PhGA  (VAS in mm) | | |  | |

CRP, C-reactive protein; PhGA, physician global assessment; SJC28, 28 swollen joint count; VAS, visual analogue scale.

**Supplementary Fig. 2B.** Matrix model of the proportion of patients with joint space narrowing >0 based on SJC28, CRP, and PhGA. Data are presented as % (95% confidence interval). Predicted probability of patients represented by shading: green, <15.0%; yellow, 15.0‒21.0%; red, >21.0%.

| SJC28 | >12 | **28.9 (22.8, 36.0)** | **30.4 (25.7, 35.5)** | **31.9 (26.6, 37.7)** | >11 | CRP (mg/L) |
| --- | --- | --- | --- | --- | --- | --- |
|  | 8–12 | **23.9 (19.1, 29.6)** | **25.2 (21.6, 29.2)** | **26.5 (22.1, 31.6)** |  |  |
|  | <8 | **19.6 (14.6, 25.7)** | **20.7 (16.2, 26.0)** | **21.8 (16.6, 28.2)** |  |  |
|  | >12 | **20.7 (16.5, 25.7)** | **21.9 (18.7, 25.4)** | **23.1 (19.1, 27.7)** | 3.5–11 |  |
|  | 8–12 | **16.8 (13.8, 20.3)** | **17.8 (15.8, 20.1)** | **18.8 (15.7, 22.5)** |  |  |
|  | <8 | **13.5 (10.4, 17.5)** | **14.3 (11.5, 17.8)** | **15.2 (11.5, 19.8)** |  |  |
|  | >12 | **14.4 (10.7, 19.0)** | **15.3 (11.9, 19.3)** | **16.2 (12.1, 21.2)** | <3.5 |  |
|  | 8–12 | **11.5 (8.8, 14.8)** | **12.2 (9.8, 15.2)** | **13.0 (9.8, 17.0)** |  |  |
|  | <8 | **9.1 (6.6, 12.4)** | **9.7 (7.2, 12.9)** | **10.3 (7.3, 14.5)** |  |  |
|  | | <50 | 50–80 | >80 |  | |
|  | | PhGA  (VAS in mm) | | |  | |

CRP, C-reactive protein; PhGA, physician global assessment; SJC28, 28 swollen joint count; VAS, visual analogue scale.

**Supplementary Fig. 3A.** Matrix model of the proportion of patients in remission or low disease activity by SDAI at week 24/30 based on SJC28, CRP, and PhGA. Data are presented as % (95% confidence interval). Predicted probability of patients in remission with low disease activity represented by shading: green, >47.0%; yellow, 40.0‒47.0%; red, <40.0%.

| SJC28 | >12 | **44.4 (37.3, 51.8)** | **40.4 (34.1, 47.0)** | **35.6 (29.7, 42.1)** | >11 | CRP (mg/L) |
| --- | --- | --- | --- | --- | --- | --- |
|  | 8–12 | **47.6 (40.4, 54.8)** | **43.5 (37.3, 49.9)** | **38.6 (32.4, 45.2** |  |  |
|  | <8 | **56.6 (49.2, 63.8)** | **52.5 (45.2, 59.8)** | **47.5 (40.1, 55.0)** |  |  |
|  | >12 | **43.9 (37.2, 50.9)** | **39.9 (33.8, 46.4)** | **35.2 (29.0, 41.9)** | 3.5–11 |  |
|  | 8–12 | **47.1 (40.5, 53.8)** | **43.0 (37.1, 49.1)** | **38.1 (31.8, 44.9)** |  |  |
|  | <8 | **56.1 (49.3, 62.7)** | **52.0 (45.1, 58.9)** | **47.0 (39.5, 54.6)** |  |  |
|  | >12 | **46.3 (39.3, 53.4)** | **42.2 (35.6, 49.1)** | **37.4 (30.7, 44.5)** | <3.5 |  |
|  | 8–12 | **49.4 (42.9, 55.9)** | **45.3 (39.3, 51.5)** | **40.4 (33.8, 47.3)** |  |  |
|  | <8 | **58.4 (51.6, 64.9)** | **54.4 (47.2, 61.4)** | **49.3 (41.6, 57.1)** |  |  |
|  | | <50 | 50–80 | >80 |  | |
|  | | PhGA  (VAS in mm) | | |  | |

CRP, C-reactive protein; SDAI, Simplified Disease Activity Index; PhGA, physician global assessment; SJC28, 28 swollen joint count; VAS, visual analogue scale.

**Supplementary Fig. 3B.** Matrix model of the proportion of patients in remission or with low disease activity by DAS28 at week 24/30 based on SJC28, CRP, and PhGA. Data are presented as % (95% confidence interval). Predicted probability of patients in remission with low disease activity represented by shading: green >32.0%; yellow, 27.0–32.0%; red, <27.0%.

| SJC28 | >12 | **24.7 (19.1, 31.3)** | **22.9 (18.0, 28.7)** | **19.9 (15.4, 25.3)** | >11 | CRP (mg/L) |
| --- | --- | --- | --- | --- | --- | --- |
|  | 8–12 | **26.4 (20.7, 33.2)** | **24.5 (19.6, 30.3)** | **21.4 (16.6, 27.0)** |  |  |
|  | <8 | **32.4 (25.8, 39.9)** | **30.2 (23.9, 37.4)** | **26.6 (20.6, 33.6)** |  |  |
|  | >12 | **29.3 (23.4, 36.0)** | **27.3 (21.9, 33.4)** | **23.9 (18.6, 30.1)** | 3.5–11 |  |
|  | 8–12 | **31.2 (25.4, 37.8)** | **29.1 (23.9, 34.9)** | **25.6 (20.2, 31.9)** |  |  |
|  | <8 | **37.7 (31.2, 44.7)** | **35.4 (29.0, 42.4)** | **31.4 (24.8, 38.9)** |  |  |
|  | >12 | **33.7 (27.2, 40.9)** | **31.5 (25.4, 38.3)** | **27.8 (21.7, 34.7)** | <3.5 |  |
|  | 8–12 | **35.7 (29.6, 42.3)** | **33.4 (27.8, 39.6)** | **29.6 (23.7, 36.3)** |  |  |
|  | <8 | **42.6 (35.8, 49.7)** | **40.1 (33.1, 47.6)** | **35.9 (28.6, 44.0)** |  |  |
|  | | <50 | 50–80 | >80 |  | |
|  | | PhGA  (VAS in mm) | | |  | |

CRP, C-reactive protein; DAS28, disease activity score by 28 joint count; PhGA, physician global assessment; SJC28, 28 swollen joint count; VAS, visual analogue scale.

**Supplementary Fig. 4.** Number of patients corresponding to each tertile of the 3 baseline factors.

| SJC28 | >12 | **41** | **65** | **111** | >11 | CRP (mg/L) |
| --- | --- | --- | --- | --- | --- | --- |
|  | 8–12 | **27** | **57** | **61** |  |  |
|  | <8 | **34** | **38** | **33** |  |  |
|  | >12 | **53** | **61** | **61** | 3.5-11 |  |
|  | 8–12 | **46** | **57** | **40** |  |  |
|  | <8 | **58** | **39** | **37** |  |  |
|  | >12 | **54** | **56** | **46** | <3.5 |  |
|  | 8–12 | **52** | **70** | **47** |  |  |
|  | <8 | **59** | **36** | **27** |  |  |
|  | | <50 | 50–80 | >80 |  | |
|  | | PhGA  (VAS in mm) | | |  | |

CRP, C-reactive protein; PhGA, physician global assessment; SJC28, 28 swollen joint count; VAS, visual analogue scale.
